# Distributional hypothesis as isomorphism between word-word co-occurrence and analogical parallelograms

**DOI:** 10.1371/journal.pone.0312151

**Published:** 2024-10-21

**Authors:** Takuma Torii, Akihiro Maeda, Shohei Hidaka

**Affiliations:** 1 Tokyo Denki University, Hatoyama, Saitama, Japan; 2 Japan Advanced Institute of Science and Technology, Nomi, Ishikawa, Japan; European Commission, ITALY

## Abstract

Most of the modern natural language processing (NLP) techniques are based on the vector space models of language, in which each word is represented by a vector in a high dimensional space. One of the earliest successes was demonstrated by the four-term analogical reasoning task: what is to C as B is to A? The trained word vectors form “parallelograms” representing the quadruple of words in analogy. This discovery in NLP offers us insight into our understanding of human semantic representation of words via analogical reasoning. Despite successful applications of the large-scale language models, it has not been fully understood why such parallelograms emerge by learning through natural language data. As the vector space model is not optimized to form parallelograms, the key structure related to geometric shapes of word vectors is expected to be in the data, rather than the models. In the present article, we test our hypothesis that such parallelogram arrangement of word vectors readily exists in the co-occurrence statistics of language. Our approach focuses more on the data itself, and it is different from the existing theoretical approach trying to find the mechanism of parallelogram formation in the algorithms and/or vector arithmetic operations on word vectors. First, our analysis suggested that analogical reasoning is possible by decomposition of the bigram co-occurrence matrix. Second, we demonstrated the formation of a parallelepiped, a more structured geometric object than a parallelogram, by creating a small artificial corpus and its word vectors. With these results, we propose a refined form of distributional hypothesis pointing out an isomorphism between a sort of symmetry or exchangeability and word co-occurrence statistics.

## 1 Introduction

### 1.1 Distributional hypothesis

‘Evolution’ of the language processing capability of machines has dramatically accelerated in the decade beginning 2012. The accuracy of machine translation has reached the human level or perhaps greater than that of educated non-native speakers. These successes of machine-learning language models have suggested how natural languages are organized.

The *distributional hypothesis* [1] considers language as an organized system that exhibits the capability of determining a class of words given the context of a word to be determined. This theoretical idea of the distributional hypothesis postulates that words that occur in similar contexts tend to have similar meanings. For example, “an apple” and “a banana” are both allowed to appear in similar contexts, e.g., “she eats ___ for breakfast” and “___ is a fruit”. However, they may not appear in similar contexts of “a bus” and “a train”, e.g., “she takes ___ home”. When we think of the fill-in-the-blank problem “John eats ___ for breakfast”, words that refer to something edible and common in breakfast would be selected by the context of the blank. The fill-in-the-blank problem, or its variants, are commonly encountered in everyday communication. For example, when a speaker cannot come up with the name of something on their mind, the speaker often instead says its attributes or properties. The listener then guesses the name of something which has those characteristics. Imagine that a child says “fruit”, “red”, “juicy”, “sour”, and so on—you may recall “apple” or “strawberry”. This phenomenon can be considered an instance of the distributional hypothesis in communication. This inference seems to be (partly) possible because of the distributional structure of our language.

### 1.2 Distributional models of language

One pervasive method of implementing the distributional hypothesis is counting the co-occurrence of words in pairs, triplets, or n-grams. Such naive co-occurrence counting has, however, a few technical issues: the combinatorial space of word pairs is too large to sample sufficiently (e.g., a bigram (pair) table of word-word co-occurrence has 10^12^ cells for 10^6^ word types), which results in the underestimation of co-occurrence probability. Thus, one needs further *compressed* representations of the co-occurrence table, in which the compressed representations hopefully preserve the distributional structure of the words in the table and the language. Latent Semantic Analysis (LSA) [2] is one of the earliest such attempts. The underlying idea of LSA is that a sparse co-occurrence matrix *M* can be approximated by vector representation of words, called *word vectors*. The transformation of a co-occurrence matrix to a word vector representation is called *word embedding*. It has been demonstrated that word vector algorithms can solve linguistic tasks, albeit that their performance was limited (see [3] for review).

More recently, Mikolov et al. [4] discovered that four-term analogy problems can be solved accurately using their artificial neural network, called *skip-gram*, which is an instance of the *word2vec* class of models. The word2vec models have become common word embedding models in recent years. Four-term analogy problem questions “what is to C as B is to A?” denoted by, A : B :: C : what. Formally, the model needs to predict word d given the triplet of query words a, b, and c. For example, the question, man : woman :: king : ___, expects the answer ‘queen’. Importantly, word2vec was not optimized to solve these four-term analogy questions, but rather to predict the context words for each target word—making it a form of the fill-in-the-blank problem. However, with learned word vectors, e.g., *v*_king_, *v*_man_, *v*_woman_, one can answer the analogy task by vector arithmetic *v*_king_ − *v*_man_ + *v*_woman_ ≈ *v*_queen_. This four-word relationship is often referred to as parallelogram [5] due to its geometric shape in the vector space. Thus, such high performance in the four-term analogy is considered a consequence of learning more general statistical properties of language. Since analogical reasoning requires not only syntactic but also semantic aspects of language, their successes in the analogy task have been viewed as strong support for the distributional hypothesis. And since analogy, at least in a strong form such as that discussed in [6], was considered to be uniquely human, this discovery had a strong impact on a variety of research fields. This discovery in NLP offers/recalls an important question to be answered from the view-point of computational linguistics and cognitive science: the four-term analogy task is a kind of semantic task, and its solutions offered by the vector space models would provide insight into our understanding of the human semantic representation of words via analogical/relational reasoning.

To solve analogy problems, word2vec needs to successfully extract latent and distributional structures of the language, which is represented in the vector form. It has not fully understood why such a parallelogram emerges by learning through natural language data. The word vector models are in general *not* optimized to form any specific arrangement of word vectors, but are rather optimized to approximate co-occurrence statistics. Since Mikolov et al. [4]’s discovery, researchers of related fields have been attracted to resolve this “mystery” of parallelogram formed in word2vec. Many NLP researchers noted the learning algorithm proposed by Mikolov et al. [4] to address a technical problem in training a word2vec model: its computation of the conditional probability distribution of ‘context words’ given a ‘center word’ is intractable when the vocabulary size is quite large. The technique proposed by Mikolov et al. [4] for this problem is called *negative sampling*, which makes this computation tractable. Since word2vec is basically an artificial neural network of typical form, most researchers of related fields consider that a new negative sampling algorithm is essential to acquire a word vector representation capable of solving analogy tasks (e.g., [7–9]). The current consensus (see, e.g., [3]) in NLP is provided by Levy, Goldberg, & Dagan [7, 10]. They formally analyzed the negative sampling algorithm and claimed that the analogy performance of word2vec could be explained as resulting from the factorization of the PPMI (positive pointwise mutual information) matrix, where PPMI is one of the most popular preprocess of the co-occurrence matrix in NLP.

In the present article, we take a distinct approach that focuses more on data rather than the model and/or leaning algorithms. Conversely, most of the existing theoretical studies described above focused on models/algorithms rather than data. We hypothesized that a parallelogram arrangement of word vectors readily exists in the co-occurrence statistics of language: in other words, the models/algorithms only mirror parallelograms presented in data but do not learn parallelograms not presented in data. Our original motivation in conducting the present research was to understand aspects of the nature of human language via the distributional hypothesis [1] connecting the syntactic and semantic spaces of our language with statistical regularity. Our hypothesis may be interpreted as a refinement of Harris’s distributional hypothesis in which we specify how co-occurrence statistics are related to semantic and syntactic aspects of language (e.g, man : king :: woman : queen, and, man : men :: king : kings, respectively).

### 1.3 What structure in the co-occurrence matrix enables analogical reasoning?

Word analogy relations emerging in word embedding have been extensively investigated in the literature. While there is abundant research into how word analogy works [8, 11–13], few studies have analyzed linguistic regularities in corpora. Chiang et al. [14] show that the major source of linguistic regularity in a corpus is *not* the direct, i.e., syntagmatic, co-occurrence information. In an effort to seek the source of statistical cues of analogy relations, Chiang et al. manipulated direct co-occurrence information by modifying the training corpus for language models, specifically by removing/replacing sentences in the original corpus that contain analogical word pairs in their analogy test set, e.g., France : Paris. They reported that analogy performance degraded only marginally. It is noteworthy that Chiang et al. [14] attempted to ascribe the parallelograms of word vectors to the co-occurrence statistics, and their finding suggests that one of major factors forming the parallelograms is likely due to across-sentence statistical regularity, rather than within-sentence co-occurrence.

In this study, we focus on the relationship between statistical regularity in a corpus and geometric patterns of word vectors learned through it. As suggested in [14], some kind of co-occurrence statistics at the sentence level would be correlated to the formation of parallelograms in word vectors. Thus, this study aims to answer the following questions: (1) are the parallelograms in word vectors formed mainly by corpus statistics, rather than specific properties of learning algorithms? And (2) if the answer to Question (1) is yes, what is the condition in the sentence level necessary to form the analogical relationship between word vectors?

To answer Question (1), we test the hypothesis that the raw co-occurrence frequency has sufficient information for the analogy task, and thus gives a minimal data set, which holds the essence of the natural corpus, and is simple enough to analyze mathematically. Although this hypothesis has been partly validated elsewhere (such as [15]), we revisit this assumption and add more empirical evidence in Section 3 and 4.

Next, to answer Question (2), we take a constructive approach in which a small artificial corpus is built and closely analyzed in Section 5. Although word-to-word direct co-occurrence can be manipulated easily in a natural corpus, it is not straightforward to manipulate sentence-by-sentence statistics, as these may reflect underlying natural contexts. With our constructive approach, we can systematically manipulate sentence level statistics and analyze their effect on the formation of parallelograms in word vector space.

Note that our goal of this corpus analysis in Section 3 is not to propose an algorithm to achieve better analogical performance, but to provide support/evidence for the distributional hypothesis directly; i.e., word co-occurrence information itself correlates with semantics via word analogy. Besides, Section 4 provides further evidence that the negative sampling algorithm is sufficient but not necessary for word analogy. These provide a justification for the theoretical analysis of raw co-occurrence structure in Section 5.

Connecting the co-occurrence matrix to analogical parallelograms directly naturally leads to a constructive approach: simulation to test which type of co-occurrence may embed a parallelogram in the word vector space. Thus, we take the two types of complementary approaches, namely data-driven analysis of the co-occurrence matrix, and constructive simulation by creating and manipulating a small corpus. In this theoretical analysis, we provide a necessary and sufficient condition to form analogical parallelopipeds in such a small corpus. While most research provides sufficient algorithms for linguistic tasks, little research provides necessary conditions for even small tasks.

In the following, we briefly introduce the word2vec model in Section 2, followed by an analysis of a co-occurrence matrix in Section 3, word2vec models without negative sampling in Section 4, and the constructive approach in Section 5. Lastly, we discuss future directions toward an understanding of the semantic nature of the underlying word co-occurrence.

## 2 word2vec and analogy

### 2.1 The word embedding algorithm

Here, we briefly introduce the key ideas of word2vec, specifically the skip-gram and the continuous bag-of-words artificial neural network architecture, as that knowledge will be required in the succeeding sections. The word2vec models consist of three layers, *n* input nodes, *d* hidden nodes, and *n* output nodes, where *n* is the vocabulary size. Initially, every word *w* in vocabulary *W* is represented by a so-called one-hot vector *e*_*w*_ of length *n*. Given a long sequence of words represented by one-hot vectors, the goal of optimization is to obtain a *d* dimensional compressed representation *v*_*w*_, called a word vector, for every word *w* ∈ *W*, where *d* ≪ *n*. Denote by *w*_*t*_ a word at the position *t* in the corpus. The skip-gram word2vec model seeks the corpus to identify every sequence (*w*_*t*−*k*_, …, *w*_*t*−1_, *w*_*t*_, *w*_*t*+1_, …, *w*_*t*+*k*_), the *k* preceding and *k* following context words around the center word *w*_*t*_. Then, the model is trained to optimize the latent word vectors {*v*_*w*_}_*w* ∈ *W*_, for each *w*_*t*_ to predict all their context words ${\vec{w}}_{t}=\left(w_{t-k},\ldots ,w_{t-1},w_{t+1},\ldots ,w_{t+k}\right)$ simultaneously throughout the corpus. Regarding the continuous bag-of-words model, this model is trained to predict the center word *w*_*t*_ given the context words. Mikolov et al. [4] defined for the word2vec models the conditional probability of occurrence *y* in the context of *x* as follows:

$$\begin{array}{c} P\left(y|x\right)=\frac{\text{exp}(v_{y}\cdot v_{x})}{\sum_{w\in W}\;\text{exp}\;\left(v_{w}\cdot v_{x}\right)}\;\text{,} \end{array}$$ (1)

where *v*_*y*_ ⋅ *v*_*x*_ is the inner product of word vectors *v*_*x*_ and *v*_*y*_.

If the vocabulary size *n* is small enough, this artificial neural network can be trained with the classic error back-propagation procedure. However, this is intractable if vocabulary size *n* is quite large. Mikolov et al. [4] therefore introduced the negative sampling algorithm to handle computation of the denominator of the above equation. Their computational experiments suggested that the skip-gram type and continuous bag-of-words type are comparable for many linguistic tasks, although computational cost of the continuous bag-of-words type is 2*k* times cheaper than that of the skip-gram type. In this paper, we will use the continuous bag-of-words type, which is the default option of the Python library Gensim.

### 2.2 Analogical reasoning procedure

Using a trained word2vec, Mikolov et al. [4] demonstrated that it can solve their four-term analogy questions. Consider, for example, the problem, man : woman :: king : ___, and the correct answer is “queen”. Given the word vectors *v*_man_, *v*_woman_, *v*_king_ for the cue words, decide the most likely word *y* by calculating the cosine similarity measure $\text{cosine}\left(v_{x},v_{y}\right)=\frac{v_{x}\cdot v_{y}}{\|v_{x}\left\|\;\right\|v_{y}\|}$ for all words *x*:

$$\begin{array}{c} v_{y}=\underset{v_{x}\;:\;x\in W\backslash A}{\text{argmax}}\;\text{cosine}\left(v_{\text{king}}-v_{\text{man}}+v_{\text{woman}},\;v_{x}\right)\;\text{,} \end{array}$$ (2)

where the set of the query words *A* = {man, woman, king} is excluded from the set of answer candidates *W*, the whole vocabulary or its subset. The response is defined correct, if *y* = queen, i.e., the word vector *v*_*y*_ is *v*_queen_. The overall percentage of correct responses is about 66% for the 19,544 questions. This hardly seems attributable to chance alone, as the probability of correct responses is by chance 1/(*n* − 3), where *n* is vocabulary size, when assuming uniformly random choices over all candidates.

If any model answers correctly for a quadruple using Eq (2), these four word vectors may have to form a parallelogram in the vector space. Indeed, Mikolov et al. [4] graphically showed the presence of parallelograms in a lower-dimensional subspace.

### 2.3 Analogy test set

The Google analogy test set was constructed by Mikolov et al. [4] to examine analogy performance, and is now commonly used as one of the benchmark problems in NLP. It contains 905 unique words and consists of a total of 19,544 problems in 14 problem categories, of which 45% are semantic (5 categories, e.g., Capital-city and Family) and 55% syntactic (9 categories, e.g., Comparative and Plural). For example, man : woman :: king :: queen in the Family category, and car : cars :: eye : eyes in the Plural category. It was systematically generated to contain every combination and permutation of word pairs in each problem category. For each problem A : B :: C : D, the set also contains its symmetric variant C : D :: A : B. Thus, achieving a very high analogy performance indicates that word vector models represent systematically both semantic and syntactic relations of words of the language.

## 3 Analogical reasoning with an almost-raw co-occurrence matrix

As introduced in Section 1.2, past studies exploring the analogical reasoning based on the word2vec or others [8–10] have essentially hypothesized and concluded that word2vec or other transformations such as PPMI is crucial to having good analogy performance. In this study, however, we hypothesize that a raw co-occurrence matrix itself or its matrix decomposition is sufficient for analogical reasoning.

### 3.1 Method

To test our hypothesis, we directly counted the frequencies of pairwise co-occurrence of all words in the English Wikipedia dump corpus wiki-english-20171001. During our preprocessing of corpus texts, metadata of the Wikipedia articles were removed and uppercase letters were changed to lowercase. The text data contains approximately 2.9 billion words, of which 7.6 million words are unique. The window size for word pair counting was *k* = 5. Although we counted them all, algebraic operations using the full co-occurrence matrix were impossible due to our limited available computational power. Hence, for the analogical task, we only used the square sub-matrices corresponding to the union of the two sets of words: (1) the 905 unique words in the Google test set, and (2) the top 1,000 (or 10,000) unique words in order of the co-occurrence frequency with the 905 words in the Google test set. This was decided in order to (1) make it possible to answer all the analogy problems in the Google test set; and because (2) the fundamental thesis of the distributional hypothesis, namely that co-occurrence statistics are informative. The resulting size of the unique words, or vocabulary, was 1487 and 10072, and so the size of the sub-matrices was 1487×1487 and 10072×10072. We call these 1487-choice and 10072-choice analogy tasks. Denote this co-occurrence matrix by $M\in R_{\geq 0}^{n\times n}$ with vocabulary size *n*.

In NLP, it is commonly recognized that application of singular-value decomposition (SVD) to the co-occurrence matrix improves performance of linguistic tasks. Indeed, this is the heart of Latent Semantic Analysis, a classical method to obtain word vectors. Technically, SVD is a decomposition of a real matrix *M* of arbitrary finite size to the form *M* = *U*Σ*V*^⊤^, where matrix *U* and *V* are real orthogonal matrices and the diagonal matrix Σ contains singular values in its diagonal elements. The number of non-zero singular values is equal to the rank of *M*. SVD is in this context commonly used to approximate and smooth *M*. By taking the first *d* columns of *U* and *d* rows of *V*^⊤^ corresponding to the top *d* largest singular values, ${\tilde{M}}_{d}=U_{d}\;\Sigma_{d}\;V_{d}^{⊤}$ approximates *M* with the *d* dimensions. By taking the first *d* dimensions, the *d* dimensional word vectors for *n* words are obtained as $U_{d}\;\Sigma_{d}^{1/2}\in R^{n\times d}$. Since the word2vec was trained to construct 300-dimensional word vectors, which was the number conventionally used in related research fields, *d* = 300 was used in this paper.

Our co-occurrence-based models, with and without singular-value decomposition, are listed in Table 1, including the neural network models that appear in this Section 3 and later in Section 4. We describe components of them in more detail in Section 3.2.

**Table 1:** List of distributional vector space models in the present paper.

Model	Word vectors
freq	Raw co-occurrence frequencies
logfreq	Logarithms of raw co-occurrence frequencies
ppmi	PPMI-transformed co-occurrence frequencies
freq-SVD	freq with SVD
logfreq-SVD	logfreq with SVD
ppmi-SVD	ppmi with SVD
word2vec-CE-topn	word2vec trained by cross-entropy error backprop with vocabulary size *n*
word2vec-NS-topn	word2vec trained by negative sampling with vocabulary size *n*
word2vec-NS-full	word2vec trained by negative sampling with the full Wikipedia corpus

Additionally, we trained our word2vec models using the sample code of the Python library Gensim [16], in which the continuous bag-of-words type and the negative sampling algorithm are specified by default. We used our own preprocessed text data, the English Wikipedia dump, described above. We call this model word2vec-NS-full after its Negative-Sampling objective. The window size *k* = 5 is the same. By the default option of Gensim, only the words that occur 100 or more times in the corpus were used for training the model. The number of unique words was approximately 0.32 million.

Since the word2vec-NS-full model was trained to represent a vocabulary of size approximately 0.32 million from the Wikipedia corpus, it is not directly comparable with other co-occurrence matrix models representing a substantially smaller vocabulary (of size, e.g., 1487 or 10072). In other words, the word2vec-NS-full was exposed to co-occurrence information more/other than that contained in a small co-occurrence matrix. Thus, more information must be compressed in the resulting word vectors of the word2vec-NS-full model. To be fair in terms of co-occurrence information, we trained the word2vec models using an simulated “corpus” composed of 500,000,000 sentences generated based on the unigram probability distribution *U*(*w*) and conditional probability distributions *P*(*c*|*w*) over a subset of the full vocabulary of the Wikipedia dump corpus (see S1 Appendix for the technical details). This intermediate simulated corpus was required for the technical reason that the Python Gensim library only supports a text stream, or a text file, as inputs. The size of a subset of the vocabulary was 1487 or 10072, depending on the 1487- or 10072-choice analogy tasks. We call these models word2vec-NS-topn.

### 3.2 Results

**Fig 1:**
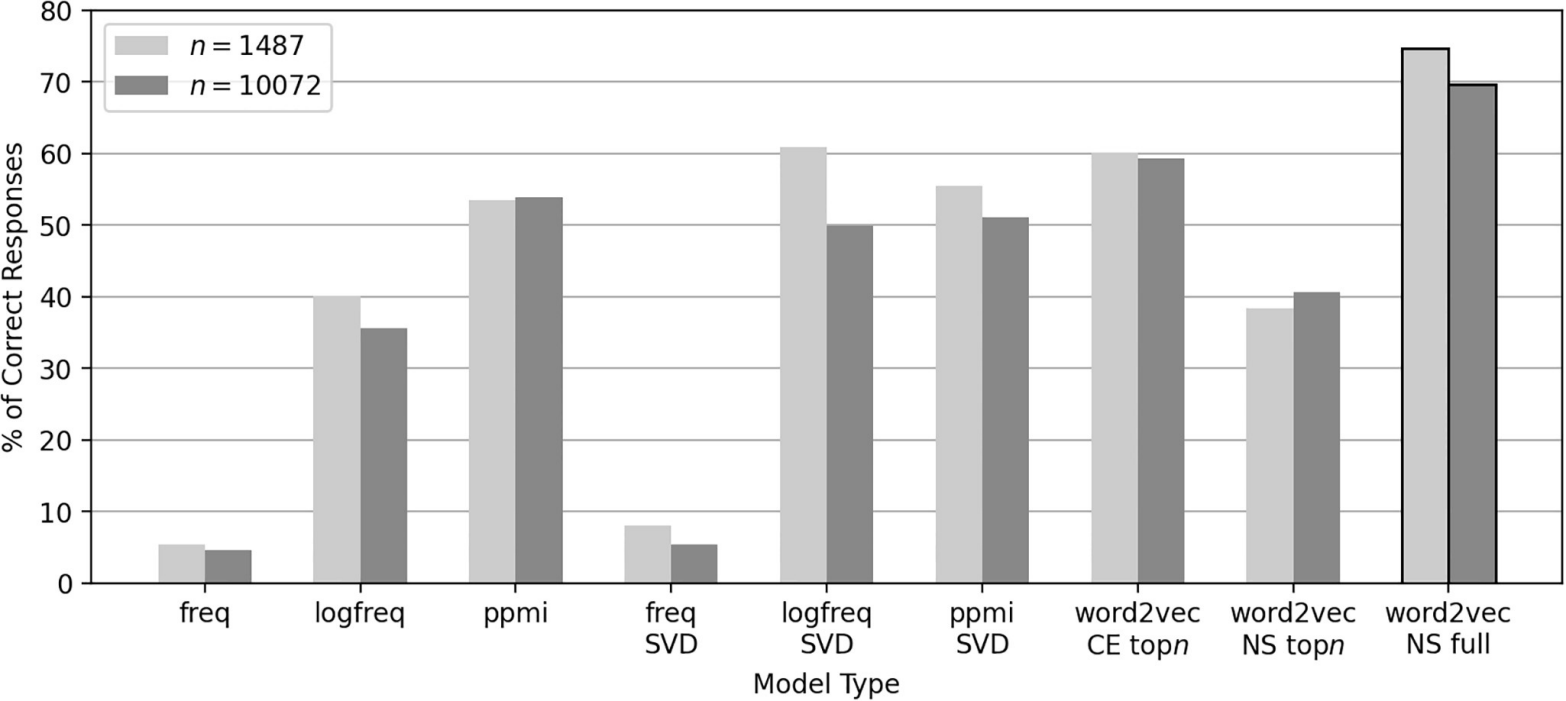
Four-term analogy performances of distributional models.

**Fig 2:**
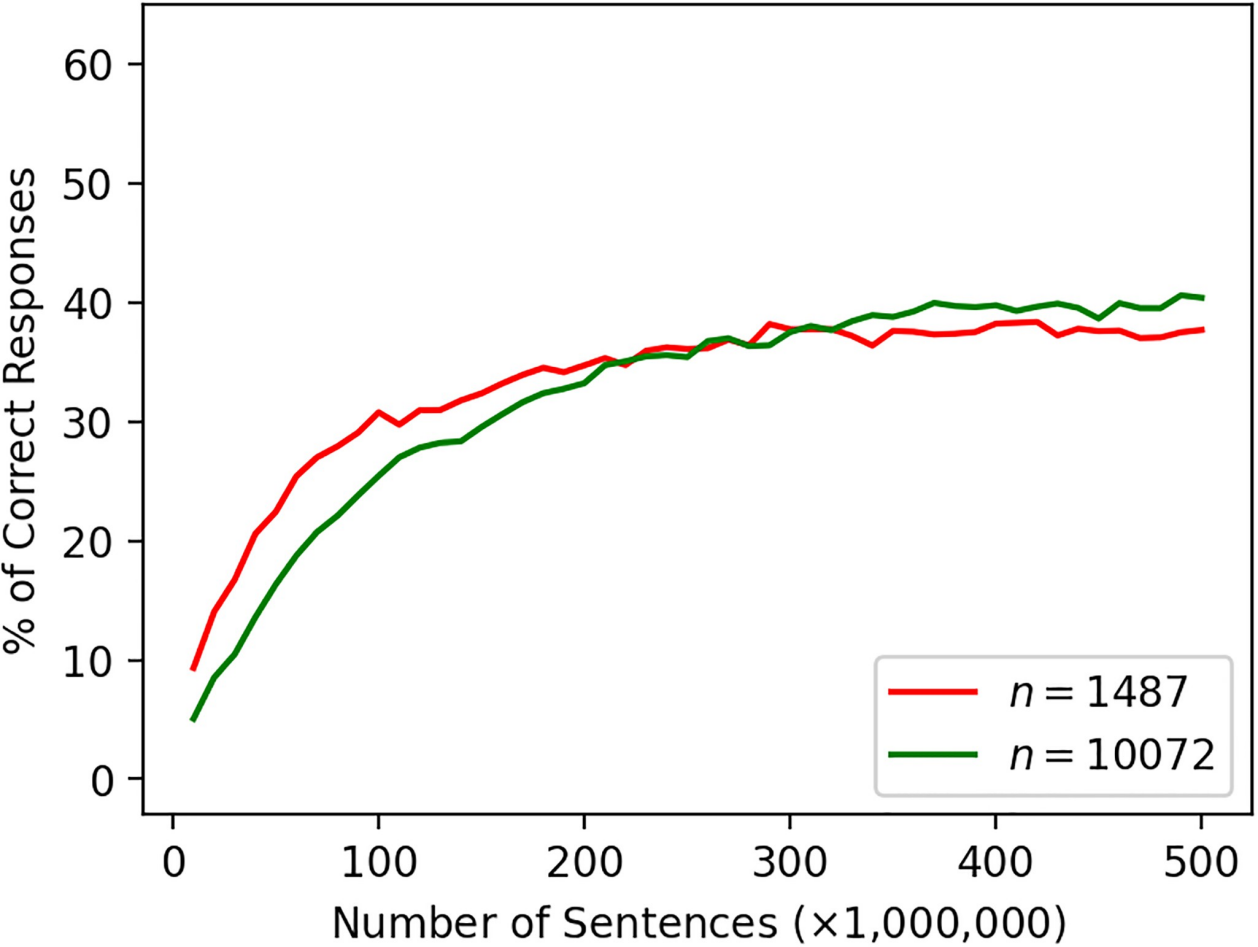
Analogy performance of the vocabulary-restricted word2vec models with negative sampling. To manipulate vocabulary size, artificial corpora were generated from the co-occurrence statistics and used for training, instead of the original corpus.

Fig 1 shows the performances for the four-term analogy task using distributional models. Performance of the word2vec-NS-full model was 75% correct responses for the 1487-choice (and 69% for the 10072-choice) analogy tasks. On the other hand, the performance of the word2vec-NS-topn model was only 38% for 1487-choice (and 40% for 10072-choice) analogy tasks (see Fig 2 for their performance curves). We treat these as benchmarks given by the negative sampling. Note that the former are not directly comparable in terms of analogy performance, since the models were trained using the whole Wikipedia dump corpus whereas the latter ones and the other co-occurrence models appearing below were constructed using only a small top-ranked subset of the vocabulary of the corpus.

For the models freq, the rows of the co-occurrence frequency matrix *M* were directly used as word vectors. The models showed accuracy below 6% (and 5%). For the models logfreq, the logarithms of the rows of *M* were used as word vectors; stated more precisely, log(max{*M*_*yx*_, 1}) for all co-occurrence frequency *M*_*yx*_ of context word *y* of center word *x*. By applying the logarithms, the performance of the model was significantly increased, by about 40% (and 35%). Similar effects have been repeatedly reported in NLP research (see, e.g., [15]). We consider the logarithm worked as a type of smoothing against Zipf’s law, which states that word frequency follows a power law and thus high-frequency words (e.g., “the”) dominate over lower-frequency words in the calculation of cosine similarity via inner products. Taking logarithm can relax this domination and so lower-frequency words can have substantial influence on calculation of the similarity of word vectors. As logfreq is comparable to word2vec-NS-topn, this partially supports our hypothesis that information required to solve linguistic tasks is inside the corpus data. However, there is room for further improvement induced by word2vec. To eliminate this possibility, we applied SVD, a classical linear word embedding method, to the log-frequency matrices of *M*. Since SVD is linear, in contrast with word2vec, which is nonlinear, it would be helpful to resolve the mystery of word2vec if we could replicate the word2vec-level performance by applying SVD only to the log-frequency matrices. Surprisingly, the performance of the logfreq-SVD models was improved above 61% (and 50%), which is markedly higher than word2vec-NS-topn albeit lower than word2vec-NS-full. This result supports the other half of our hypothesis, namely that information required for four-term analogies resides primarily inside the text data. Finally, the performances of the ppmi and ppmi-SVD were about 54% (and 54%) and 55% (and 51%).

We note a few things here. (1) The performance of co-occurrence matrix (+ linear embedding) models, such as logfreq-SVD and ppmi-SVD, is no greater than that of the word2vec-NS-full models. Accordingly, these results do not eliminate the possibility that the negative sampling algorithm at the real-scale vocabulary corpus can find a much better word vector representation in practice. (2) Comparing the performance of ppmi-SVD and word2vec-NS-topn, our results suggest that Levy, Goldberg, & Dagan [7]’s claim that the negative sampling is mostly PPMI does not appear to be the case in the present setting.

### 3.3 Discussion

#### 3.3.1 Why the decomposed co-occurrence matrix is close to word2vec

If the originally formulated word2vec models were successfully trained, the word (and context) vectors $V_{0},V_{1}^{⊤}\in R^{n\times d}$ determine the conditional co-occurrence probability matrix *P*(*y*|*x*) in Eq (1). By taking logarithm, $V_{0}\;V_{1}^{⊤}\in R^{n\times n}$ is extracted (the normalizing term is ignored), and thus the skip-gram model could be viewed as an approximate matrix decomposition of the form $V_{0}\;V_{1}^{⊤}\approx \tilde{M}$ for unknown $\tilde{M}$. Given the results above, this suggests that “up-to-rank-*d* matrix decomposition of the logarithm of *M*” is essentially what the word2vec models do. This hypothesis differs from a previous study [7], which concluded that word2vec is equivalent to the PPMI-like smoothing, or a matrix decomposition of the PPMI-smoothed matrix of *M*. Our model, namely word2vec as a co-occurrence matrix decomposition, can be viewed as one of the simplest and most straightforward implementations of the distributional hypothesis [1].

#### 3.3.2 Related works

We remark that the logfreq-SVD model that combines logarithmic normalization with singular value decomposition is not new. We believe that similar analyses had been conducted repeatedly. However, since the task evaluation heavily depends on the characteristics of the corpora analyzed, we believe that the present work may augment existing findings and/or provide something new, in particular with regard to research aimed at characterizing the latent distributional structure of language.

Indeed, Tian et al. [15] examined the Google analogy test performance by applying SVD to the logarithmic co-occurrence matrix. They reported that their ln-SVD model achieved 52% in the Google test set. Unlike our case, the goal of Tian et al. [15]’s analysis was to examine their theory of additive composition, in which they theoretically justified the use of logarithmic normalization and singular value decomposition. However, a weakness of their analysis is that they had to ignore approximately 45% of the Google test set, due to a limitation in the acquired vocabulary they used from the British National Corpus, with 100 million tokens. On the other hand, we used the English Wikipedia dump 2017 corpus, with 2.9 billion tokens, and importantly the resulting co-occurrence statistics contain all 905 unique words appear in the Google analogy test set. Thus, we achieved performance evaluation for the whole problem set. Although there were differences in preparation of the co-occurrence matrices, and in coverage of the test problems, our results seem consistent with those of [15].

## 4 Analogical reasoning with word2vec w/o negative sampling

If the negative sampling algorithm is essential, i.e., necessary, for high-performance word embedding for analogy tasks, word2vec models without negative sampling should show unacceptably low performance. In part of the previous section, we obtained results which did not support this by analyzing an almost-raw co-occurrence matrix as a language model. In this section, we continue to pursue the specific goal above by directly training word2vec models (i.e., neural networks) with a classical error back-propagation instead of the modern negative sampling. If the negative sampling is unnecessary, word2vec models with cross-entropy error back-propagation should perform as well as other models.

### 4.1 Neural network training

We used the co-occurrence matrix constructed from the Wikipedia corpus to train our plain word2vec model, instead of scanning throughout the corpus. Since the corpus is huge, the co-occurrence matrix gives a good estimate of the joint and conditional co-occurrence probability distributions. Using the whole co-occurrence matrix is again computationally intractable, and hence we only used the top-ranked *n* = 1487 words in the 1487-choice (or *n* = 10072 for the 10072-choice) analogy tasks. Then, as originally formulated, a word2vec artificial neural network was trained to approximate the conditional probability distributions *P*(*c*|*w*) calculated from the co-occurrence matrix *M* of size *n*. The cross-entropy error is defined as its objective function and for each *w* the element-wise errors are back-propagated. Center words *w* follow probability distribution over *n* words, *P*(*w*) ∝ *U*(*w*)^*α*^, where *U*(*w*) is the unigram word frequency distribution and 0 ≤ *α* ≤ 1 is a normalizing parameter. Since 905 words among *n* words appear in the analogy test set, the neural network must be trained for almost all those words. We call these models word2vec-CE-topn after their Cross-Entropy objective.

Choosing a smaller *α* increases the probability of observing low-frequency words in contrast to high-frequency words. Mikolov et al. [4] for example introduced a similar trick in a different context for choosing a good negative sampling distribution, and noted that their trick actually works to obtain better word representations.

### 4.2 Results

**Fig 3:**
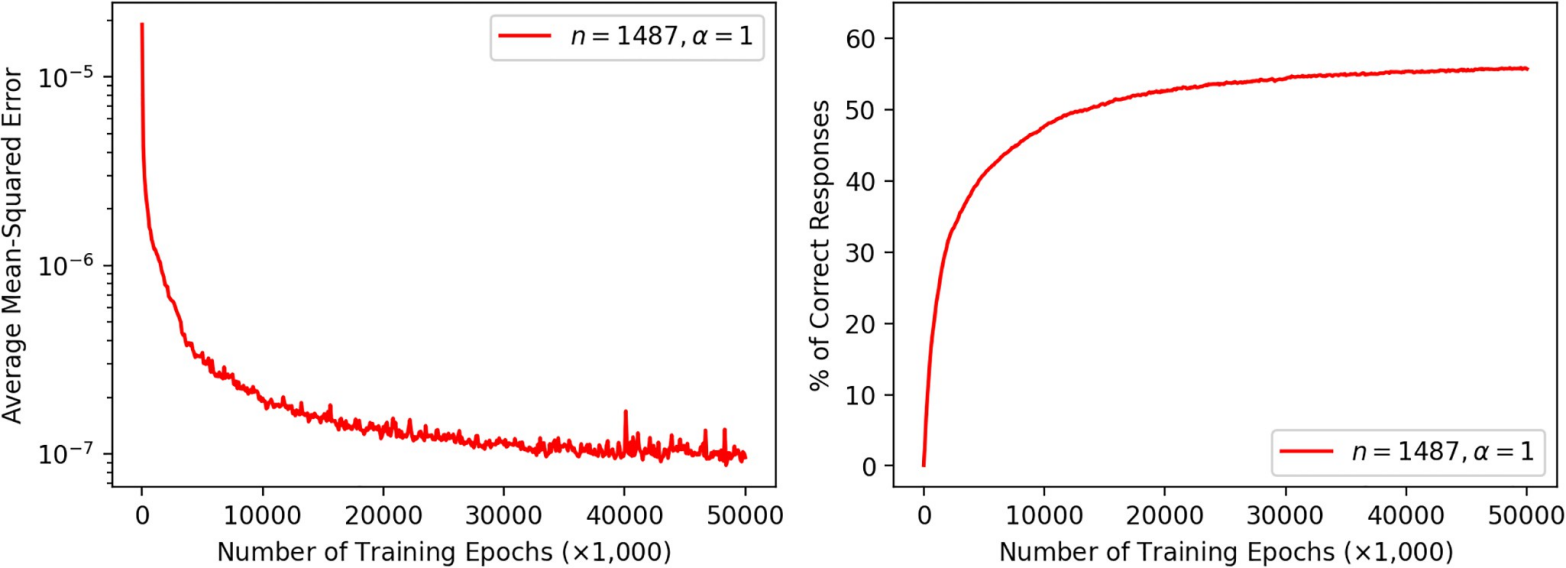
Learning curve and analogy performance of a word2vec model trained with back-propagation.

First, we trained a model with *n* = 1487 and *α* = 1 over 50,000,000 words from *P*(*w*). The learning curves in terms of the mean-squared prediction error and analogy performance are shown in Fig 3. The average (mean-squared) prediction errors gradually decreased and converged to below the level 10^−7^, and until the end, the analogy performance of this model slowly increased to become largely saturated. The eventual performance of this model for the 1487-choice analogy task was approximately 56%, which is markedly higher than the chance level and comparable to many other models in Fig 1.

**Fig 4:**
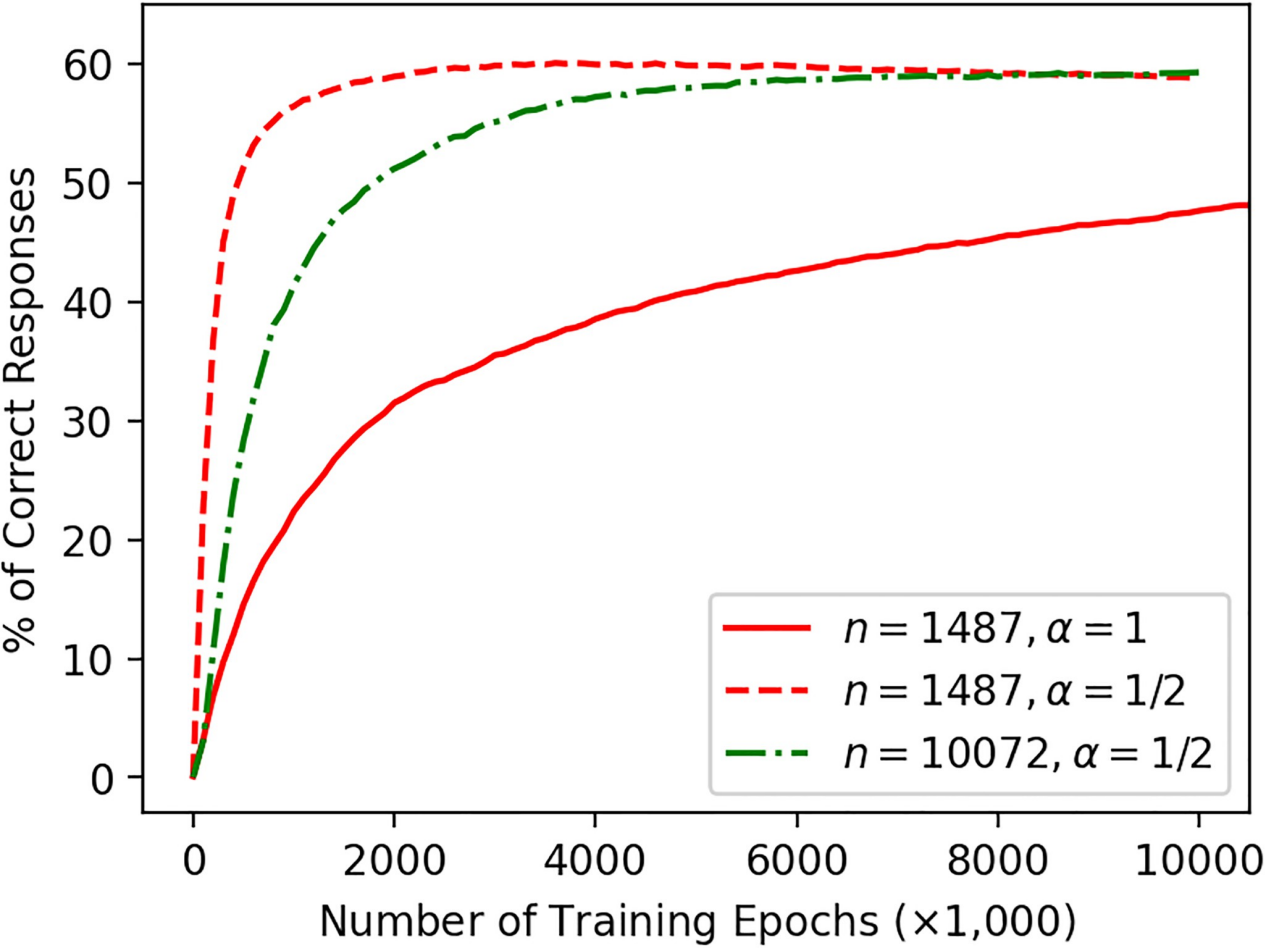
Analogy performance of a word2vec model trained with back-propagation.

Second, we trained a model with *n* = 1487 and *α* = 1/2 over 10,000,000 words. Compared with the first model with *α* = 1, this second model increased analogy performance more rapidly and achieved the highest performance approximately 60% after only 3, 600, 000 exposures to words (see Fig 4). Thus, the normalizing parameter *α* seems to work to more rapidly acquire equally-good-performance word vectors with a small number of word exposures. This simple empirical fact motivated us to train the third model with a larger vocabulary size.

Third, we trained a model with *n* = 10072 and *α* = 1/2 over 10,000,000 words. Since neural network training when *n* = 10072 was intractably computationally expensive, we could not train the model with *α* = 1. We assume that the word representation acquired with *α* = 1 is as good as that with *α* = 1/2 in respect to the four-term analogy test. With *α* = 1/2, analogy performance of the model was again eventually approximately 59%, comparable to the *n* = 1487 model, after 10,000,000 exposures (see Fig 4).

### 4.3 Discussion

All these results suggest that the negative sampling algorithm is not essential to obtaining word vectors suitable for analogy tasks. In other words, without negative sampling, the embedded word vectors can solve analogy problems fairly well. In the previous section, the performance of the word2vec-NS-topn models, trained using the negative sampling for a *n* = 1487 or *n* = 10072 restricted artificial corpus, was about 40%. Thus, the error back-propagation algorithm, or the word2vec-CE-topn models, can give better word embedding than the negative sampling at least if the vocabulary size is tractably small. Importantly, the error back-propagation algorithm directly optimized the original objective function of the word2vec models. In contrast, negative sampling was introduced as an computational trick by Mikolov et al. [4], and Levy, Goldberg, & Dagan [7] analyzed it as a substantial optimization trick. Our results here suggest that such a computational trick is not guaranteed to produce high-performance word representation.

As seen in the previous section, it should be noted that the word2vec-NS-full models, trained with negative sampling throughout the full Wikipedia corpus, actually achieves the highest performance—75%—among all the models. No evidence to elucidate the ultimate reason for this observation is currently available. There are at least two factors: the negative sampling and the vocabulary size. These two factors can interact. We postulate that vocabulary size is the primary, although testing this hypothesis is computationally hard. In any case, this observation tells us that the negative sampling algorithm is actually effective in obtaining favorable word embedding in various applications, regardless of whether it is necessary or not, especially when the vocabulary size is quite large, as this makes the optimization problem computationally tractable.

## 5 Constructive approach to the parallelograms

The analysis in Section 3 suggests that there is a subspace of the co-occurrence matrix in which a parallelogram is formed by a particular set of word vectors, as each word may have multiple aspects. For example, “king” is more similar with “queen” on the Royalty axis, but more similar with “man” on the Sex axis. Such a multi-aspect structure of the word “king” is considered captured by a parallelepiped, rather than a parallelogram. Although each analogy question tests a parallelogram, a collection of analogy questions would test a parallelepiped, or a more complex geometric object.

In this section, we take a constructive approach to address how this parallelepiped structure is involved with the syntactic or semantic nature of a language. Specifically, we construct a small toy corpus, which forms an idealized parallelepiped structure among the word vectors, and analyze what conditions would be essential to form some parallelepiped of word vectors.

### 5.1 Goal

The main goal of this case study is to identify the underlying relationship between a word generator system and word vector space, with a special focus on sentence-level statistical regularity. In this study, the word generator is supposed to be a Markov process that generates a word according to a certain conditional probability distribution given the previous context words. Our analysis will address, in this toy corpus, what kind of sentence-level statistical structure in such a word generator is isomorphic to a parallelepiped of the word vectors.

### 5.2 Premise

The empirical analysis reported in Section 3 and 4 suggests that the word vectors defined by the log of the co-occurrence matrix or its low dimensional projection by singular value decomposition is sufficient to form a parallelogram related to analogical reasoning. This finding justifies the following analysis of word vectors consisting of raw co-occurrence counts, which are the simplest form of word vectors.

### 5.3 Toy corpus

**Fig 5:**
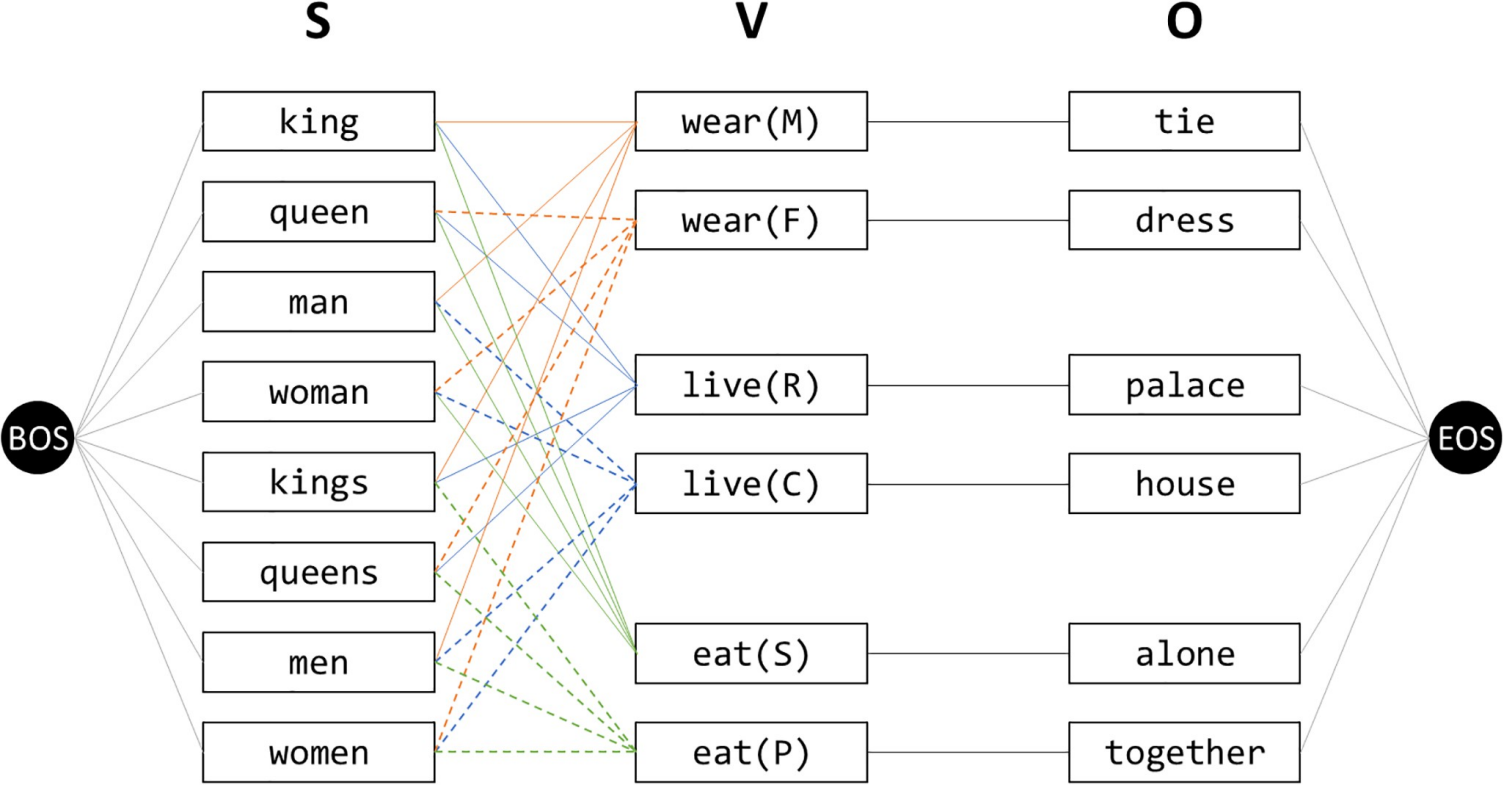
A hidden Markov model generating the 24 sentences in the toy corpus. Any hidden state other than the verbs generates the word with probability 1. For example, the state “king” generates the word “king”. On the other hand, the two hidden states for each verb generate the same. For example, both states “live (R)” and “live (C)” generate the word “live”. “BOS”: Beginning of Sentence, “EOS”: End of Sentence.

We created a corpus of 24 artificial sentences which are not strictly grammatical, but have a minimal syntactic and semantic structure. Each of the sentences in this corpus consists of three words in the form of Subject-Verb-Object (S-V-O), such as “king live palace”. Fig 5 depicts possible S-V-O routes by line segments. The corpus consists of 17 words, consisting of 8 subjects, 3 verbs, and 6 objects. Among the maximum possible 8 × 3 × 6 = 144 sentences, only 24 = 8 × 3 sentences can be generated, which implicitly represents the hypothetical semantic relationship between the underlying concepts that these words refer to. The point of our design is the 3 verbs shown in Fig 5 with their latent states in parentheses: (M) for male, (F) for female, (R) for royal, (C) for civil, (S) for single, and (P) for plural. Pairs of these latent states form 3 aspects: (M) and (F) for Sex, (R) and (C) for Royalty, and (S) and (P) for Number.

#### 5.3.1 Word embedding with uniform sentence probabilities

**Fig 6:**
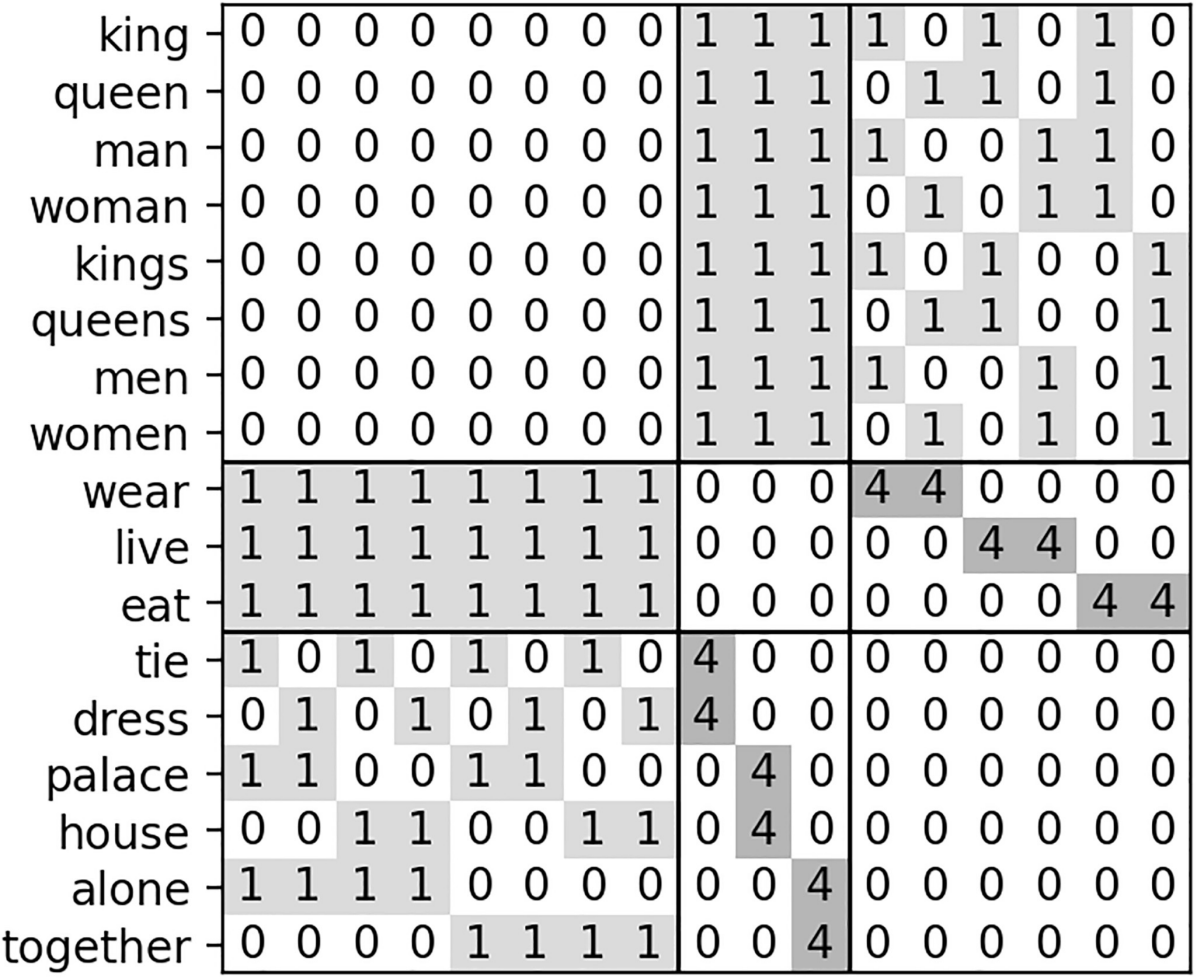
The co-occurrence matrix generated with the uniform sentence probability distribution. The numbers in the cells represent their relative frequency.

**Fig 7:**
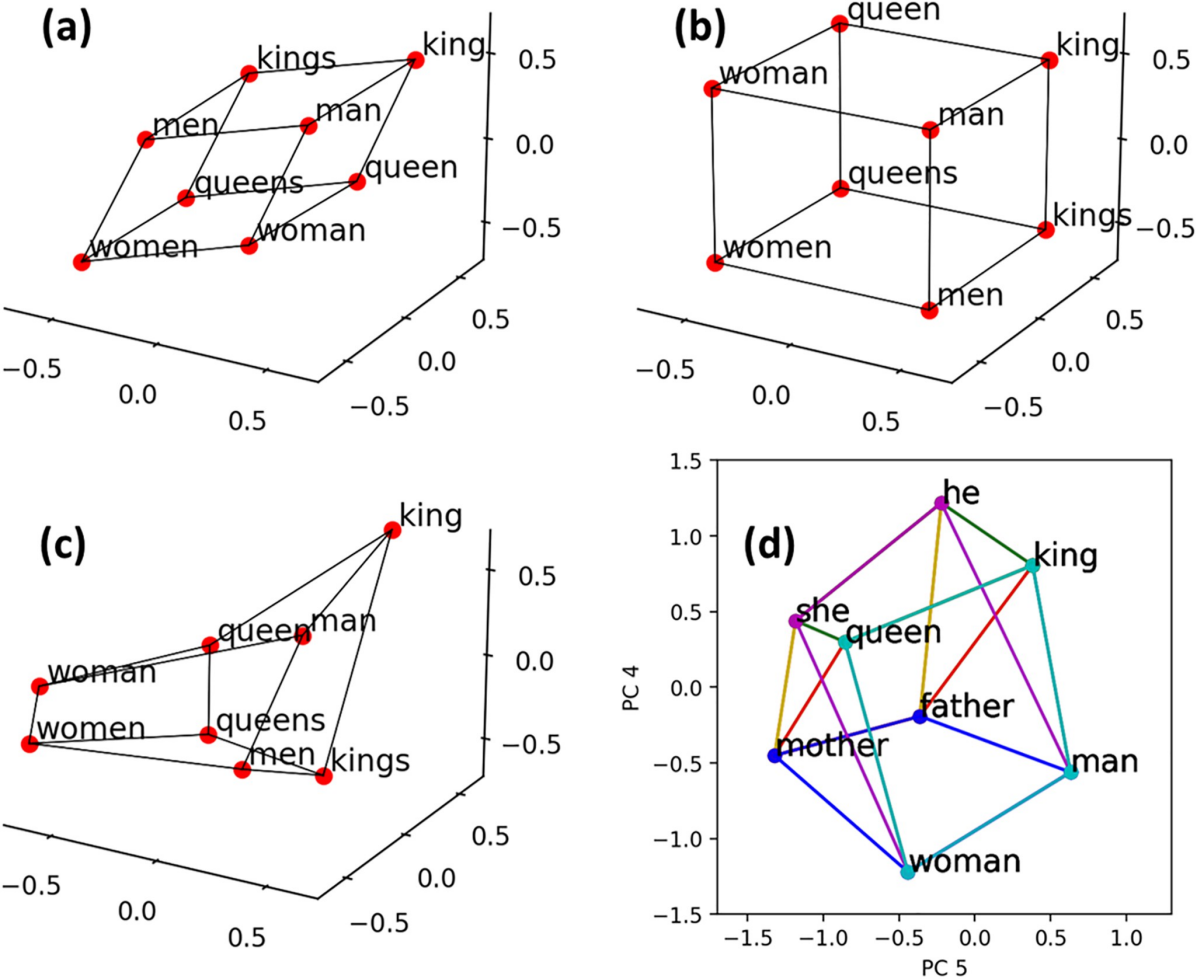
(Non-)parallelepipeds embedded in the co-occurrence matrix of (a) uniform toy corpus (b) with orthogonal basis and (c) non-uniform toy corpus. (d) Parallelepiped embedded in the co-occurrence matrix of a natural corpus.

First, we analyzed the co-occurrence matrix constructed for the toy corpus with each of the sentences generated with the equal probability 1/24. We used the same co-occurrence counting scheme with a window size *k* = 5, which is in this case equivalent to treating all of the words in every single sentence as co-occurrence. In this case, the co-occurrence matrix *C*, up to scale and permutation similarity, can be written with the two block matrices $C_{0}\in R^{8\times 9}$ and $C_{1}\in R^{9\times 9}$ by

$$\begin{array}{c} C=(\begin{array}{ccc} 0_{8,8} & 1_{8,3} & C_{0}\\ 1_{3,8} & 0_{3,3} & C_{1}\\ C_{0}^{⊤} & C_{1}^{⊤} & 0_{6,6} \end{array})\;\text{,} \end{array}$$ (3)

where *C*_1_ = 4*I*_3_ ⊗ **1**_1,2_ and ⊗ denotes the Kronecker product. Fig 6 illustrates this co-occurrence matrix, Eq 3. Now to think about the parallelogram relationship, consider the sub-matrix, denoted *C*, composed of the first 8 row vectors, corresponding to the 8 subject words. This matrix *C* has the rank 4, and it lives in a 3 dimensional affine space. Namely, there are some linearly independent affine basis vectors $b_{0},b_{1},b_{2},b_{3}\in R^{9}$ such that $C=A\;{\left(b_{1},b_{2},b_{3}\right)}^{⊤}+1_{8}\;b_{0}^{⊤}$ with a unique matrix $A\in R^{8\times 3}$. Let $C^{\prime }≔C\;\left(I_{9}-\frac{1}{9}1_{9,9}\right)$, and choose *B* ≔ (*b*_1_, *b*_2_, *b*_3_) with $b_{1},b_{2},b_{3}\in R^{9}$ from non-zero row vectors of *C*′. Then, the three dimensional coordinates of the 8 points, i.e., *A*, are given by the row vectors of *C*′*B*(*B*^⊤^*B*)^−1^, in which a “parallelepiped” is embedded, as shown in Fig 7(a). Thus, this uniform toy corpus gives a sufficient condition or the existence of a way of embedding a parallelepiped in the co-occurrence matrix. Instead of this basis, consider the orthogonal basis (*b*_1_, *b*_2_, *b*_3_) for *B* such that each basis vector corresponds to one of the three aspects Sex, Royalty, and Number by assigning ±1 for their corresponding two object words (e.g., use *b*_1_ for Sex by setting *b*_1,tie_ = + 1 and *b*_1,dress_ = −1). Embedded word vectors appear to be a parallelepiped as shown in Fig 7(b). This word embedding extracted the three latent aspects of the verbs: the embedded parallelepiped is composed of the 8 subjects in the three dimensions, each representing one of the Sex, Royalty, and Number aspects.

#### 5.3.2 Symmetry breaker against parallelepiped: Non-uniform sentence probabilities

It is also important to demonstrate on which condition the parallelepiped embedded in a co-occurrence matrix is *broken*, as such a demonstration gives a necessary condition for the parallelepiped formation. To do so, we consider a variation of the toy corpus, called the non-uniform toy corpus, in which each probability *p*_*i*_ to sample the *i*^th^ sentence is randomly assigned from the uniform distribution over [0, 1]. Fig 7(c) shows the same set of the 8 word vectors visualized in the same way as in Fig 7(a), for a set of non-uniform random probabilities *p*_*i*_. These 8 word vectors form neither a parallelepiped nor parallelograms. As the only difference between the uniform and non-uniform toy corpus is their sampling probability, this result suggests that a certain symmetric relationship in the probability distributions is needed to hold the parallelepiped.

This demonstrates that some sentence-level statistical regularity is necessary to hold a parallelepiped, even if within-sentence word-word co-occurrence is fixed. Remember that empirical analysis [14] removing word-to-word co-occurrence suggests across-sentence statistical regularity. Our toy corpus has some qualitatively consistent structure with this past finding.

### 5.4 A parallelepiped in natural co-occurrence

The demonstration with the toy corpus above suggests that a certain class of word vectors will form a parallelepiped relationship, if the class of word vectors show independent syntactic-semantic statistical regularities on its word usage. We test this prediction by searching whether such a parallelepiped exists for a class of word vectors embedded in a natural co-occurrence matrix (logfreq-SVD, size 1487). Fig 7(d) shows an example of a parallelopiped-like object composed of word vectors corresponding to eight words in the Family category of the Google test set. It is visualized in a two-dimensional subspace obtained by performing principal component analysis of a data matrix of size 48×1487 representing the 1487-dimensional word vectors for all 48 unique words in the Family category. This confirms our prediction. We emphasize that it merely demonstrates the “existence” of a parallelopiped. The formation of parallelopiped-like objects in high-dimensional spaces is technically hard to characterize and so itself requires further investigation.

### 5.5 Necessary and sufficient conditions for a parallelepiped in co-occurrence matrix

The requirement of the uniform sentence probability 1/24 may be too strong. In this subsection, we relax this requirement and find a necessary and sufficient condition for the co-occurrence matrix from the toy corpus to form a parallelepiped.

First, let us define a parallelepiped as follows.

**Definition 1**. *Let* $C≔{\left(\begin{array}{cccc} c_{1}^{⊤} & c_{2}^{⊤} & \cdots & c_{8}^{⊤} \end{array}\right)}^{⊤}\in R^{8\times d}$ *be the matrix with the row vectors* $c_{1},c_{2},\ldots ,c_{8}\in R^{1\times d}$. *The matrix* *C* *with 8 row vectors is said* *parallelepiped* *if it has some permutation (i.e., automorphism of a set) p* : {1, 2, …, 8} → {1, 2, …, 8} *and satisfies*

$$\begin{array}{c} \left\{ \begin{array}{c} c_{p(1)}-c_{p(2)}=c_{p(3)}-c_{p(4)}=c_{p(5)}-c_{p(6)}=c_{p(7)}-c_{p(8)}\\ c_{p(1)}-c_{p(3)}=c_{p(2)}-c_{p(4)}=c_{p(5)}-c_{p(6)}=c_{p(7)}-c_{p(8)}\\ c_{p(1)}-c_{p(5)}=c_{p(2)}-c_{p(6)}=c_{p(3)}-c_{p(7)}=c_{p(4)}-c_{p(8)} \end{array}\;\text{.}\right. \end{array}$$ (4)

With this definition of a class of parallelepiped, the following theorem states the necessary and sufficient condition to have this class in the co-occurrence matrix.

**Fig 8:**
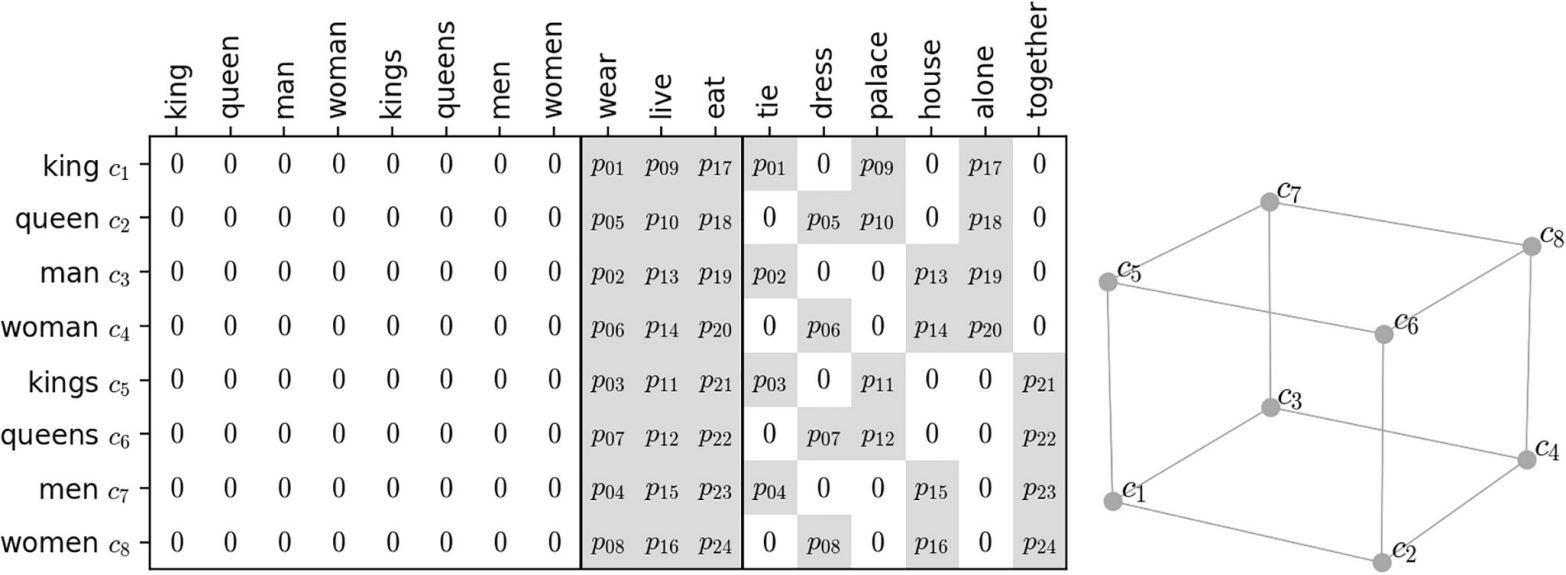
The co-occurrence matrix generated with the arbitrary sentence probability distribution. The numbers in the cells represent their probability. The row vectors are illustrated in a low dimensional subspace.

**Theorem 1**. *Given a sentence probability distribution* (*p*_01_, *p*_02_, *p*_03_, …, *p*_24_) *for the toy corpus, in* Fig 5, *comprised of 8 subject, 3 verb, and 6 object words, the resulting co-occurrence matrix in* Fig 8 *of size* 8 × 17 *in terms of the 8 subject words forms a parallelepiped if and only if the sentence probability vector* (*p*_01_, *p*_02_, *p*_03_, …, *p*_24_) *satisfies*

$$\begin{array}{c} \left\{ \begin{array}{c} p_{01}=p_{02}=p_{03}=p_{04}\\ p_{05}=p_{06}=p_{07}=p_{08}\\ p_{09}=p_{10}=p_{11}=p_{12}\\ p_{13}=p_{14}=p_{15}=p_{16}\\ p_{17}=p_{18}=p_{19}=p_{20}\\ p_{21}=p_{22}=p_{23}=p_{24} \end{array}\;\text{.}\right. \end{array}$$ (5)

We describe this in details and provide a proof below.

Denote by *p*_*i*_ the probability of generating the *i*th sentence. The numbering of the 24 sentences is arbitrary, and so we choose the numbering system illustrated in Fig 8 in the form of a co-occurrence matrix. For example, the sentence “king wear tie” is assigned number 1, whose probability is *p*_01_. Since in this corpus, every subject-object pair uniquely determines one of the 3 verbs, and thus one of the 24 sentences, the resulting co-occurrence matrix can be described in such a simple form, as shown in Fig 8, as a function of the sentence probability distribution (*p*_01_, *p*_02_, …, *p*_24_). For brevity, we reuse the symbol *C* to denote the matrix in Fig 8 composed of the row vectors for the 8 subject words, and denote by *c*_*j*_ the *j*th row vector of $C\in R^{8\times 9}$.

*Proof*. Let $C≔{\left(\begin{array}{cccc} c_{1}^{⊤} & c_{2}^{⊤} & \cdots & c_{8}^{⊤} \end{array}\right)}^{⊤}\in R^{8\times 9}$ be the matrix with the row vectors $c_{1},c_{2},\ldots ,c_{8}\in R^{1\times 9}$. Each of these row vectors contains co-occurrence counts for the 3 verbs and 6 objects, as depicted in Fig 8. Note that each *c*_*i*_ is originally a row vector in the space $R^{17}$, but the frequency count for the first 8 dimension is zero, and thus neglected without loss of generality. In the special case that *p* is the identity map, the necessary condition for the set of these eight vectors to form a parallelepiped in the space $R^{9}$ is given by Definition 1:

$$\begin{array}{c} \left\{ \begin{array}{c} c_{1}-c_{2}=c_{3}-c_{4}=c_{5}-c_{6}=c_{7}-c_{8}\\ c_{1}-c_{3}=c_{2}-c_{4}=c_{5}-c_{7}=c_{6}-c_{8}\\ c_{1}-c_{5}=c_{2}-c_{6}=c_{3}-c_{7}=c_{4}-c_{8} \end{array}\;\text{.}\right. \end{array}$$ (6)

Eq (6) with nine equalities (e.g., three equations *c*_1_ − *c*_2_ = *c*_3_ − *c*_4_, *c*_1_ − *c*_2_ = *c*_5_ − *c*_6_, and *c*_1_ − *c*_2_ = *c*_7_ − *c*_8_ in the first row) is expressed by a matrix product

$$\begin{array}{c} NC=0_{9,9}\;\text{,} \end{array}$$ (7)

where *N* is a coefficient matrix defined by

$$\begin{array}{c} N≔\left(\begin{array}{cccccccc} -1 & 1 & 1 & -1 & 0 & 0 & 0 & 0\\ -1 & 1 & 0 & 0 & 1 & -1 & 0 & 0\\ -1 & 1 & 0 & 0 & 0 & 0 & 1 & -1\\ -1 & 1 & 1 & -1 & 0 & 0 & 0 & 0\\ -1 & 0 & 1 & 0 & 1 & 0 & -1 & 0\\ -1 & 0 & 1 & 0 & 0 & 1 & 0 & -1\\ -1 & 1 & 0 & 0 & 1 & -1 & 0 & 0\\ -1 & 0 & 1 & 0 & 1 & 0 & -1 & 0\\ -1 & 0 & 0 & 1 & 1 & 0 & 0 & -1 \end{array}\right)\;\text{.} \end{array}$$ (8)

Note that, for each permutation (automorphism) *p* : {1, …, 8} → {1, …, 8}, replacing (*c*_1_, *c*_2_, …, *c*_8_) with (*c*_*p*(1)_, *c*_*p*(2)_, …, *c*_*p*(8)_) in Eq (6) gives another part of the necessary condition. Thus, the full necessary condition for *C* to form a parallelepiped is to satisfy

$$\begin{array}{c} NPC=0_{9,9}\;\text{,} \end{array}$$ (9)

for some permutation matrix *P* ∈ {0, 1}^8×8^.

Any column vector of the solution matrix *C* for Eq (9) needs to be in the kernel of the matrix *N*, $\text{ker}\;N≔\{x\in R^{8}|Nx=0_{9}\}$. The rank of this kernel is 4, and it is specifically spanned by a basis with the vectors *k*_0_, *k*_1_, *k*_2_, *k*_3_ ∈ {0, 1}^8^ defined by

$$\begin{array}{c} K≔\left(k_{0},k_{1},k_{2},k_{3}\right)≔\left(\begin{array}{cccc} 1 & 1 & 1 & 1\\ 1 & 0 & 1 & 1\\ 1 & 1 & 0 & 1\\ 1 & 0 & 0 & 1\\ 1 & 1 & 1 & 0\\ 1 & 0 & 1 & 0\\ 1 & 1 & 0 & 0\\ 1 & 0 & 0 & 0 \end{array}\right)\;\text{.} \end{array}$$ (10)

Find *NK* = **0**_9,9_ and the set of the vectors *k*_0_, *k*_1_, *k*_2_, *k*_3_ is linearly independent. To have *C* as a solution of Eq (9), every column vector of *PC* needs to be a linear combination of the vectors *k*_0_, *k*_1_, *k*_2_, *k*_3_. To see this, decompose the matrix *C* by

$$\begin{array}{c} C=\sum_{i=1}^{6}v_{i}\;u_{i}^{⊤}=\left(v_{1},\ldots ,v_{6}\right){\left(u_{1},\ldots ,u_{6}\right)}^{⊤}, \end{array}$$ (11)

where (*v*_1_, *v*_2_, *v*_3_, *v*_4_, *v*_5_, *v*_6_) are the last 6 column vectors of *C* in Fig 8, i.e.,

$$\begin{array}{cc} & V≔\left(v_{1},v_{2},v_{3},v_{4},v_{5},v_{6}\right)≔\left(\begin{array}{cccccc} p_{01} & 0 & p_{09} & 0 & p_{17} & 0\\ 0 & p_{05} & p_{10} & 0 & p_{18} & 0\\ p_{02} & 0 & 0 & p_{13} & p_{19} & 0\\ 0 & p_{06} & 0 & p_{14} & p_{20} & 0\\ p_{03} & 0 & p_{11} & 0 & 0 & p_{21}\\ 0 & p_{07} & p_{12} & 0 & 0 & p_{22}\\ p_{04} & 0 & 0 & p_{15} & 0 & p_{23}\\ 0 & p_{08} & 0 & p_{16} & 0 & p_{24} \end{array}\right) \end{array}$$ (12)

and

$$\begin{array}{cc} & U≔\left(u_{1},u_{2},u_{3},u_{4},u_{5},u_{6}\right)≔\left(\begin{array}{cccccc} 1 & 1 & 0 & 0 & 0 & 0\\ 0 & 0 & 1 & 1 & 0 & 0\\ 0 & 0 & 0 & 0 & 1 & 1\\ 1 & 0 & 0 & 0 & 0 & 0\\ 0 & 1 & 0 & 0 & 0 & 0\\ 0 & 0 & 1 & 0 & 0 & 0\\ 0 & 0 & 0 & 1 & 0 & 0\\ 0 & 0 & 0 & 0 & 1 & 0\\ 0 & 0 & 0 & 0 & 0 & 1 \end{array}\right)\;\text{.} \end{array}$$ (13)

This decomposition implies that each of the vectors *v*_1_, …, *v*_6_ needs to be a linear combination of *k*_0_, *k*_1_, *k*_2_, *k*_3_. Find the six identities with diagonal matrices *D*_1_, …, *D*_6_,

$$\begin{array}{c} \left\{ \begin{array}{c} v_{1}=D_{1}\;k_{1}\\ v_{2}=D_{2}\;\left(k_{0}-k_{1}\right)\\ v_{3}=D_{3}\;k_{2}\\ v_{4}=D_{4}\;\left(k_{0}-k_{2}\right)\\ v_{5}=D_{5}\;k_{3}\\ v_{6}=D_{6}\;\left(k_{0}-k_{3}\right) \end{array}\right. \end{array}$$ (14)

with $D_{i}≔\left(\begin{array}{ccc} v_{i,1} & & \\ & \ddots & \\ & & v_{i,6} \end{array}\right)$. Thus, for every *i* ∈ {1, …, 6}, it imposes that there is some common permutation matrix *P* ∈ {0, 1}^8×8^ with a pair of integer *j* ∈ {1, …, 3} and constant b∈R such that

$$\begin{array}{c} Pv_{i}=bk_{j}\;\;\text{or}\;\;Pv_{i}=b\;\left(k_{0}-k_{j}\right)\;\text{.} \end{array}$$ (15)

Thus, Eq (15) imposes Eq (5) for any *P*.

Next, we derive the necessary condition on *P*. The condition in Eq (15) implies

$$\begin{array}{c} V=KB\;\text{,} \end{array}$$ (15)

where $B≔\left(\begin{array}{cccccc} 0 & b_{1} & 0 & b_{2} & 0 & b_{3}\\ b_{1} & -b_{1} & 0 & 0 & 0 & 0\\ 0 & 0 & b_{2} & -b_{2} & 0 & 0\\ 0 & 0 & 0 & 0 & b_{3} & -b_{3} \end{array}\right)$. As every column vector of the matrix *PV* with some permutation matrix *P* is a linear combination of *k*_0_, *k*_1_, *k*_2_, *k*_3_ (or equivalently, a permutation *P* keeps the kernel, i.e., ker *PKB* = ker *KB*), there is a matrix $A\in R^{4\times 6}$ such that *KA* = *PV* = *PKB*. Such *A* is uniquely decided by *A* = (*K*^⊤^*K*)^−1^*K*^⊤^*PKB*, as (*K*^⊤^*K*) is invertible. Thus, we have

$$\begin{array}{c} PKB=K{\left(K^{⊤}K\right)}^{-1}K^{⊤}PKB\;\text{.} \end{array}$$ (17)

With this, there are essentially four distinct cases of necessary condition on *P*, as follows.

1. If *BB*^⊤^ is invertible or rank(*B*) = 4, we have further
  $$\begin{array}{c} PK=K{\left(K^{⊤}K\right)}^{-1}K^{⊤}PK\;\text{.} \end{array}$$ (18)
  With this, the solution is Eq (5), and there are 48 such permutations, including the identity above, that form the dihedral group.
2. If rank(*B*) = 3, a special case of Eq (5) is the solution that forms a parallelogram. For example, with a permutation matrix *P* corresponding with the permutation *p* : (1, 2, …, 8) ↦ (5, 6, 3, 4, 1, 2, 7, 8), a solution needs *p*_20_ = *p*_24_ = 0 in addition to Eq (5). In this case, the set of all the above permutations *P* forms a proper subgroup of the dihedral group.
3. If rank(*B*) = 2, a further special case of Eq (5) is the second class solution above, which forms a line segment. For example, with a permutation matrix *P* corresponding with the permutation *p* : (1, 2, …, 8) ↦ (3, 6, 5, 4, 7, 2, 1, 8), a solution needs *p*_12_ = *p*_16_ = *p*_20_ = *p*_24_ = 0 in addition to Eq (5). In this case, the set of all the above permutations *P* forms a proper subgroup of the permutation group of the class 2.
4. Otherwise, *p*_*i*_ = 0 for any integer 1 ≤ *i* ≤ 24 is the solution.

Among all the cases above, class 1 is most general, and class 4 does not satisfy the assumption that *p*_*i*_ is a probability that holds $\sum_{i=1}^{24}p_{i}=1$. Thus, class 4 with Eq (5) is the necessary condition for the set of vectors *c*_1_, *c*_2_, …, *c*_8_ to form a parallelepiped. The converse, the sufficiency of the condition in Eq (5), is already confirmed as noted above by observing that there exists some group of permutation matrices *P* for class 1, 2, and 3.

### 5.6 Implication of the mathematical analysis of co-occurrence matrix

This theorem, together with its proof, reveals the core structure of the co-occurrence matrix characterized by the dihedral group, which corresponds to the set of affine automorphisms for the parallelepiped. Eq (5) implies that the locally unigram-like structure is embedded in the bigram co-occurrence matrix *C*, if and only if the eight word vectors form a parallelepiped. In sum, Theorem 1 identifies isomorphism between distributional symmetry across sentence probabilities, with Eq (5) in the word generator and parallelepiped, and Eq (6) in word vector space. Our analysis on the toy corpus identified sentence-level statistical regularity, as suggested in [14]. This represents a locally uniform probability distribution over a certain subset of sentences, i.e., Eq (5).

Although this result is limited to our toy corpus only with 24 possible sentences, it also has an implication for more general cases. First, the rigorous result of locally uniform probability, Eq (5), may not hold for a realistic corpus, as there is no solution for Eq (6) with noisy word vectors. Instead, we can replace Eq (6) with minimization of some error function ∑_*i*,*j*_ ‖*c*_*i*_−*c*_*j*_‖^2^, and expect a similar relationship with Eq (5) in a large empirical corpus.

Second, our technique used in the proof—the kernel of the co-occurrence matrix for a given constraint by the parallelepiped—can be useful for the analysis of other kinds of geometric objects. The logic based on the kernel is not limited to our small corpus, but a general large matrix. Thus, the key question is how we can identify a latent geometric nature of an unknown set of word vectors with no prior knowledge of its underlying structure.

Third, with regard to this question, the underlying group structure may play a key role in identifying such latent geometric structures. Remember that our analysis showed that the co-occurrence matrix *C* in our case study is characterized by a dihedral group. This observation may be used in the reverse order—we can start with a potential group structure to characterize an underlying nature of word vectors in the co-occurrence matrix, and identify a geometric feature such as Eq (6).

## 6 Discussion

Previously, Levy, Goldberg, & Dagan [7] suggested that, in our interpretation, the negative sampling algorithm works as an information-theoretic pre-processing, and is thus the major factor by which the word2vec models are capable of performing analogical reasoning in terms of the four-term analogy test set. Thus, in our understanding, they considered that they provided an answer to the mystery of the analogy performance of the word2vec models by connecting the negative sampling algorithm to the PPMI transformation.

In the present paper, we revisited this suggestion by taking a distinct approach with the hypothesis that a major factor related to the formation analogical parallelograms of word vectors in a high-dimensional space can be essentially explained by the natural co-occurrence structure. Namely, a sort of the distributional hypothesis [1] plays the primary role in forming geometric shapes of word vectors, while the negative sampling algorithm, and/or PPMI transformation, provides only a secondary/auxiliary role to represent it. Our hypothesis may sound trivial —garbage in, garbage-out— in an extreme case. A more appropriate question is to what extent the almost-raw co-occurrence matrix is informative, and if is informative, how can such geometric structure of word vectors be embedded. Thus, we first confirmed that the almost-raw co-occurrence matrix is sufficiently informative to embedded a geometric structure among word vectors in Section 3 and 4. Second, we then demonstrated the construction of a geometric structure of word vectors in a co-occurrence matrix in Section 5.

In Section 3, we compared performance of the four-term analogy test among word vector models, including our logfreq model that simply uses the logarithms of the co-occurrence frequencies as word vectors. We treated this model as the simplest implementation of the distributional hypothesis. We showed that our logfreq model achieves 41% and logfreq-SVD model achieves 60% correct responses in the four-term analogy test. The latter is greatly higher than the 40% performance of the word2vec-NS-topn model, which was trained using negative sampling with the restricted but identical vocabulary appearing in other co-occurrence matrix models. We believe that this is the evidence to support our hypothesis that the distributional hypothesis is primary and the negative sampling is secondary. A similar result to our logfreq-SVD had been obtained by [15], who used a co-occurrence matrix from the British National Corpus and evaluated word analogy performance using approximately 55% of the problems in the Google test set.

In Section 4, to obtain additional evidence, we trained an artificial neural network with back-propagation, i.e., without negative sampling. The neural network, which was trained to approximate a conditional co-occurrence probability matrix, achieved 60% correct responses in the analogy test. We consider this to be the second piece of evidence supporting our hypothesis that negative sampling may not be the primary source of geometric formation of word vectors.

Our evidence suggests that the distributional structure or co-occurrence statistics of languages may have sufficient information to predict a target word from its context, as is postulated by Harris [1]. This in turn indicates that scientists should primarily focus their investigations on not the algorithm but the corpus data. Although various word embedding algorithms have been proposed since Latent Semantic Indexing [2] and importantly shown to work in practical linguistic tasks, our scientific understanding of the organization or structure of languages, or even vocabulary in simple cases, still seems poor. We considered that Mikolov et al. [4]’s discovery of parallelograms in trained word vectors with natural corpora opened a door to grasping the organization of real-size vocabularies using the language of geometry. However, we feel that less research has proceeded in this direction, as many or most researchers in artificial intelligence and machine learning have focused on algorithms rather than the structure of the natural corpus. We wanted to see progress in the direction of the geometry of language, even allowing that methods for applying the language of geometry to analyze the organization of vocabulary in natural corpora still appeared immature.

This motivated us, in Section 5, to take a constructive approach to the geometry of words as an extension of the parallelogram as an instance of the four-word relationship. We further investigated when a parallelepiped, a 3-dimensional generalization of a parallelogram, can be ready-made or already embedded in the co-occurrence matrix. Our approach is constructive, i.e., we seek the conditions for a geometric object by manipulating an artificial corpus as a source of the co-occurrence matrix. Specifically, in this paper we manipulated a probability distribution over all possible three-term S-V-O sentences. We identified necessary and sufficient conditions for sentence probabilities that the generated co-occurrence matrix embeds a parallelepiped relationship among words. This condition suggests a symmetric structure in sentence probability distribution, which may be called *local unigram*: namely, some part of bigram co-occurrence matrix is constrained to be a unigram-like structure, in sense that sampling probability of a certain class of words is independent of a certain range of previous words.

One of implications of symmetry in word vectors to human language processing is that some language structure (e.g., parallelograms formed by word vectors) may be formed through cognitive selection over time. As shown in Section 5, the parallelogram reflects underlying symmetry in the language, and such symmetry enables us to encode the language data in a more economic manner (i.e., symmetric structure copies one part of the data to another, and thus it can be encoded concisely). This would allow a language and its learners to co-evolve over generations of language learners [17, 18]—learners’ cognitive limitation constrains language structure to be learnable with a smaller computational resource, and a well-formed language enables its learner to predict new words, as demonstrated by analogical reasoning.

Our mathematical analysis in Section 5 describes a mechanism for how sentence-level symmetry in the word generator would form a geometric structure such as parallelepiped in word vector space. Partially at least, this analysis provides an insight to understanding how across-sentence probabilities contribute to the shape of word vectors, which Chiang et al. [14] have suggested by their empirical data analyses.

At least some aspect of this constructive analysis may be generalizable to other cases with various types of geometric structure embedded in larger vector spaces. We believe that this constructive analysis may open a new way to analyze, say, distributional geometry of natural corpora. Extraction of geometric objects in lossy and noisy natural corpora requires the development of statistical and robust tools for analysis. Our present constructive and geometric analysis of an artificial corpus may facilitate the development of such tools.

Finally, we should remark that our results do not rule out the possibility that the negative sampling algorithm is actually effective in practice. Evidence supporting this possibility continues to increase (see [19] and followers). This possibility does not contradict our claim that the distributional hypothesis is primary. To augment our claim, it is necessary to show in some way that equal-level analogy performance can be achieved without negative sampling or any other tricky techniques, albeit that the hurdle of computational intractability appears difficult to overcome. Regarding negative sampling, the fact that the more vocabulary the models have to learn the better their analogy performance serves as an interesting new mystery in terms of language acquisition.

## 7 Conclusion

This study attempts to provide a theoretical account of the meaning of the parallelogram in the vector space model. Our analysis of the co-occurrence matrix suggests that a type of co-occurrence matrix decomposition can provide such a parallelogram that will be useful for analogical reasoning. This empirical observation suggests that the distributional structure of languages may have sufficient information to allow a target word to be predicted from its context, as postulated by Harris [1]. This led us to a constructive approach to building a toy corpus that may or may not embed a parallelepiped in the co-occurrence matrix. This numerical simulation suggests that the parallelepiped is tightly related to a certain class of sentence probability distribution, which is less restricted than uniform but more restricted than arbitrary. We provided necessary and sufficient conditions for the sentence probability distribution to form a parallelepiped in a co-occurrence matrix. This geometric approach opens the door to grasping the organization of real-size vocabularies in the language of geometry.

## Supporting information

S1 Appendix Negative sampling given word co-occurrence statistics. (PDF)
