# Supplementary material for: Distributional hypothesis as isomorphism between word-word co-occurrence and analogical parallelograms
Source: PLoS One. 2024 Oct 21;19(10):e0312151. doi: 10.1371/journal.pone.0312151 (PMC11493305; doi:10.1371/journal.pone.0312151)
Supplement: S1 Appendix — (PDF) [file pone.0312151.s001.pdf]

A APPENDIX A. Negative sampling given word co-occurrence statistics

We adopted a Gensim Word2Vec (with negative sampling) implementation to obtain word vectors trained for a conditional co-occurrence probability matrix $P(c|w)$ and unigram probability distribution $U(w)$. Commonly, the constructor of the Word2Vec class takes an actual corpus file as input; instead of this, for our purpose, we provided a dummy, i.e., a corpus-like sentence series that contained sentences of length exactly $2k+1$, where k is the window size, whose center words are sampled from $w \sim U(\cdot)$ and the (non-center) other $2k$ words in each sentence from $c \sim P(\cdot|w)$. Thus, as expected, with k-skip 2-gram counting, the co-occurrence probability distributions approaches P and the unigram probability distribution approaches U in a very long run.
